# Merkel cell carcinoma: Clinicopathological analysis of three patients and literature review

**DOI:** 10.1515/biol-2025-1168

**Published:** 2025-11-07

**Authors:** Ting Xu, Xue Meng, Shuai Luo, Yao Li, Jinjing Wang

**Affiliations:** Department of Pathology, Zhejiang Provincial People’s Hospital Bijie Hospital, Bijie, Guizhou, P.R. China; Department of Pathology, Affiliated Hospital of Zunyi Medical University, Zunyi, Guizhou, P.R. China

**Keywords:** skin tumor, Merkel cell carcinoma, neuroendocrine carcinoma, clinicopathological features, differential diagnosis

## Abstract

To investigate the clinicopathological features of three patients with Merkel cell carcinoma (MCC). The clinicopathological features, immunophenotypes, diagnosis and differential diagnosis, treatment, and prognosis of the three patients with MCC were analyzed retrospectively. Among the three patients, two were male and one was female. The age range of the patients was 55–79 years, while their mean age was 66.6 years. The maximum and mean tumor diameters were 1.8–2.5 cm and 2.1 cm, respectively. The tumors were located in surface areas such as the face and forearm. The tumor masses were mostly round, with a gray, solid appearance and qualitative sections. Microscopic examination revealed that the MCCs of the three patients had roughly the same morphology. Light microscopy indicated that the MCCs appeared as an intradermal mass with a narrow “Grenz band” separated from the epidermis, often accompanied by necrosis (apoptotic bodies) and patchy lymphocyte infiltration. Histological assessment of the MCCs showed monomorphic cell hyperplasia with cables, trabeculae, or sheet formation, hyperchromatic nuclei, vacuoles, cytoplasm with a “salt and pepper” appearance, and nuclear division. Immunophenotyping found tumor cells that were CK (+), CD56 (+), Syn (+), EMA (+), β-catenin (membrane +), LCA (−), CD99 (−), S100 (−), HMB-45 (−), SOX-10 (−), TTF-1 (−), CDX-2 (−), and CD34 (−), along with a Ki-67 proliferation index of 60–70%. Of the three patients with MCC, two were immunophenotypic CgA negative and one was positive. Additionally, two immunophenotypes exhibited CK20 negativity, and one showed paranuclear punctate positivity for CK20. MCC is a highly malignant cutaneous neuroendocrine carcinoma, requiring the combination of pathological morphology examination and immunophenotyping to confirm the diagnosis. Moreover, the primary MCC treatment of surgical treatment should be supplemented with chemotherapy and/or local radiotherapy to alleviate its poor prognosis, easy recurrence or metastasis, and high mortality.

## 1 Background

Merkel cell carcinoma (MCC) is a rare neuroendocrine carcinoma that occurs in the skin. MCC was originally described by Toker in 1972 as a dermal trabecular carcinoma [1]. However, the origin of MCC is still debated, with some authors suggesting that it originates from Merkel cells [2]. Merkel cells were initially identified by Friedrich Sigmund Merkel in 1875 as non-dendritic, non-keratinizing epidermal “tactile cells” that function as tactile skin receptors [3]. However, a few authors have proposed that MCC is derived from primitive pluripotent stem cells capable of differentiating into keratinocytes or neuroendocrine cells [4]. Further studies have hypothesized that MCC may develop from anterior B cells based on the expression of early B-cell antigens in MCCs [5]. MCC is prevalent in the older White population and is predominant among male individuals [6]. In contrast, this skin cancer is rare in the Asian population. MCC is a highly aggressive cutaneous neuroendocrine tumor with local, regional, and distant metastases [7]. Here, three patients with MCC were reported, and their clinical pathological characteristics, immunophenotypes, diagnosis and differential diagnosis, treatment, and prognosis were assessed, aiming to improve the awareness of this rare and highly malignant tumor among clinical and pathologists.

## 2 Materials and methods

### 2.1 Clinical information and diagnosis

From 2017 to 2023, three patients were diagnosed with MCC in the Department of Pathology, Affiliated Hospital of Zunyi Medical University. Among them, two patients were male and one was female, with an average age of 66.6 years. The MCCs occurred with a clinical manifestation of a painless subcutaneous mass, which increased rapidly over a short period. In particular, one patient had a skin rupture but no pain, while another patient exhibited a skin rupture with pain. All three patients underwent local extended surgical resection and sentinel lymph node biopsy. Two pathologists reviewed all sections and confirmed the diagnosis.


**Informed consent:** Informed consent has been obtained from all individuals included in this study.
**Ethical approval:** The research related to human use has been complied with all the relevant national regulations and institutional policies and in accordance with the tenets of the Helsinki Declaration and has been approved by the Ethics Committee of Zunyi Medical University Affiliated Hospital.

### 2.2 Methods

All specimens were fixed in 10% neutral formalin and routinely dehydrated. After paraffin embedding, 3- to 4-μm-thick sections were prepared and stained with HE. Immunohistochemical staining was performed according to an EnVision two-step procedure following the kit instructions. The antibodies utilized in the staining process, including CK, CK20, CD34, SMA, HMB 45, SOX-10, S-100, p40, EMA, β-catenin, Syn, CD56, CgA, LCA, CD99, TTF-1, CDX-2, and Ki-67, were purchased from Fuzhou.

## 3 Results

### 3.1 Clinical features

**Figure 1:**
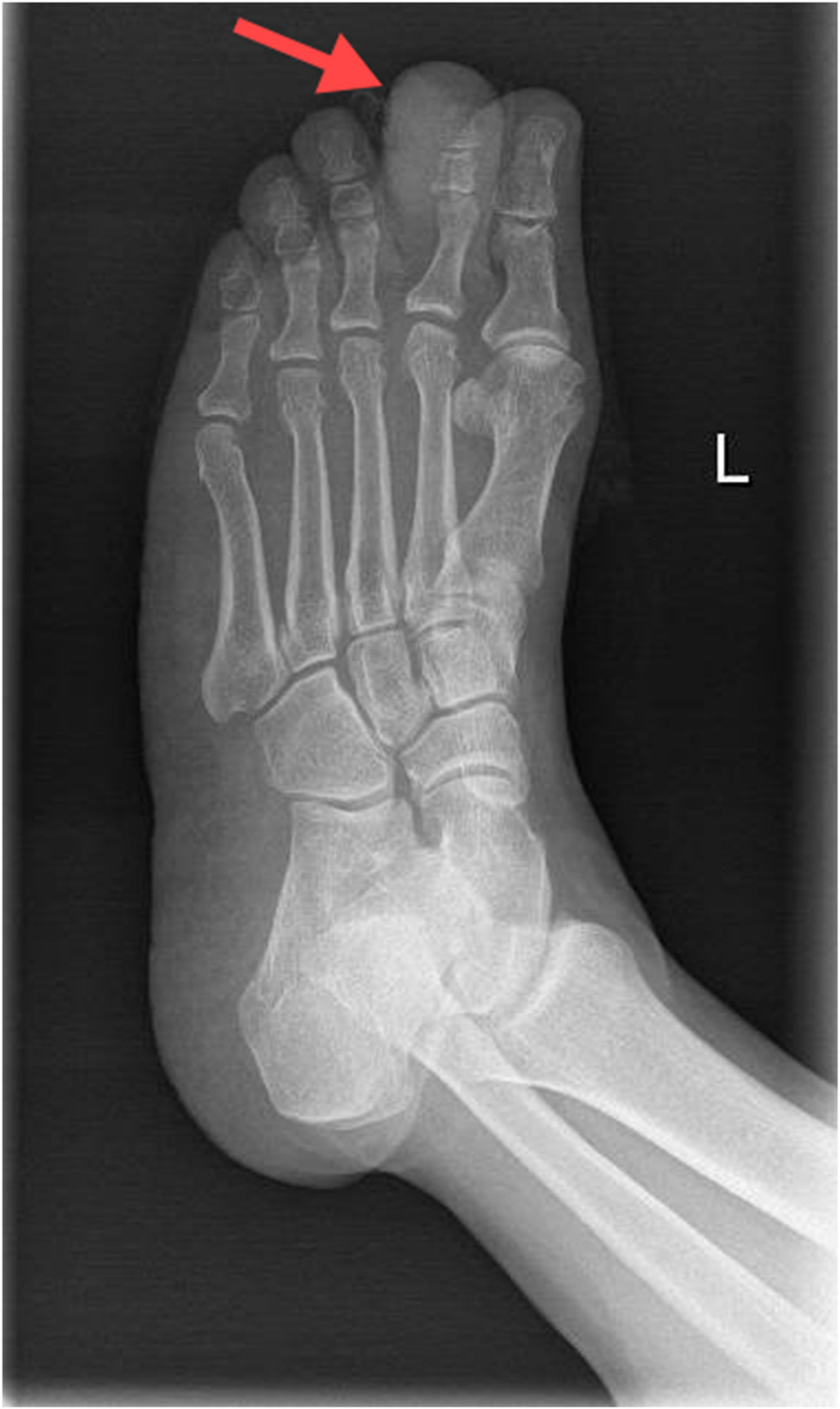
X-ray showed local bone destruction and soft tissue swelling in the distal phalanx of the second toe of the left foot in case 2.

The three patients with MCC comprised two males and one female, with ages ranging from 54 to 79 years and a mean age of 66.6 years. The tumor occurrence sites were on the second toe of the left foot (Figure 1), left forearm, and left eyelid. Clinical manifestations in one patient involved a painless subcutaneous mass, wherein the growth of the mass demonstrated prominent short-term enlargement. The remaining two patients experienced skin rupture from 2 months to 2 years. Furthermore, the preliminary clinical diagnosis of the three patients was lipoma, malignant melanoma, and squamous cell carcinoma. The treatment included local extended surgical resection and the second toe of the left foot MCC. Follow-up from 14 to 84 months revealed that all three patients had died. Table 1 provides detailed clinical information on the three patients.

**Table 1:** Clinicopathological features of 3 cases of MCC

Number	Gender	Age/year	Location	Tumor size (cm)	Methods of treatment	Follow-up
1	Female	79	Left orbit	2.5 × 2.5 × 2.0	Surgical resection	Died 84 months later
2	Male	55	Second toe of the left foot	1.8 × 0.8 × 0.6	Surgical resection	Died 15 months later
3	Male	66	Left forearm	2.0 × 1.7 × 0.8	Surgical resection	Died 14 months later

### 3.2 Pathological diagnosis

The maximum tumor diameter of the three patients ranged from 1.8 to 2.5 cm, and the average diameter was 2.1 cm. Additionally, the tumor sections appeared gray and white.

**Figure 2:**
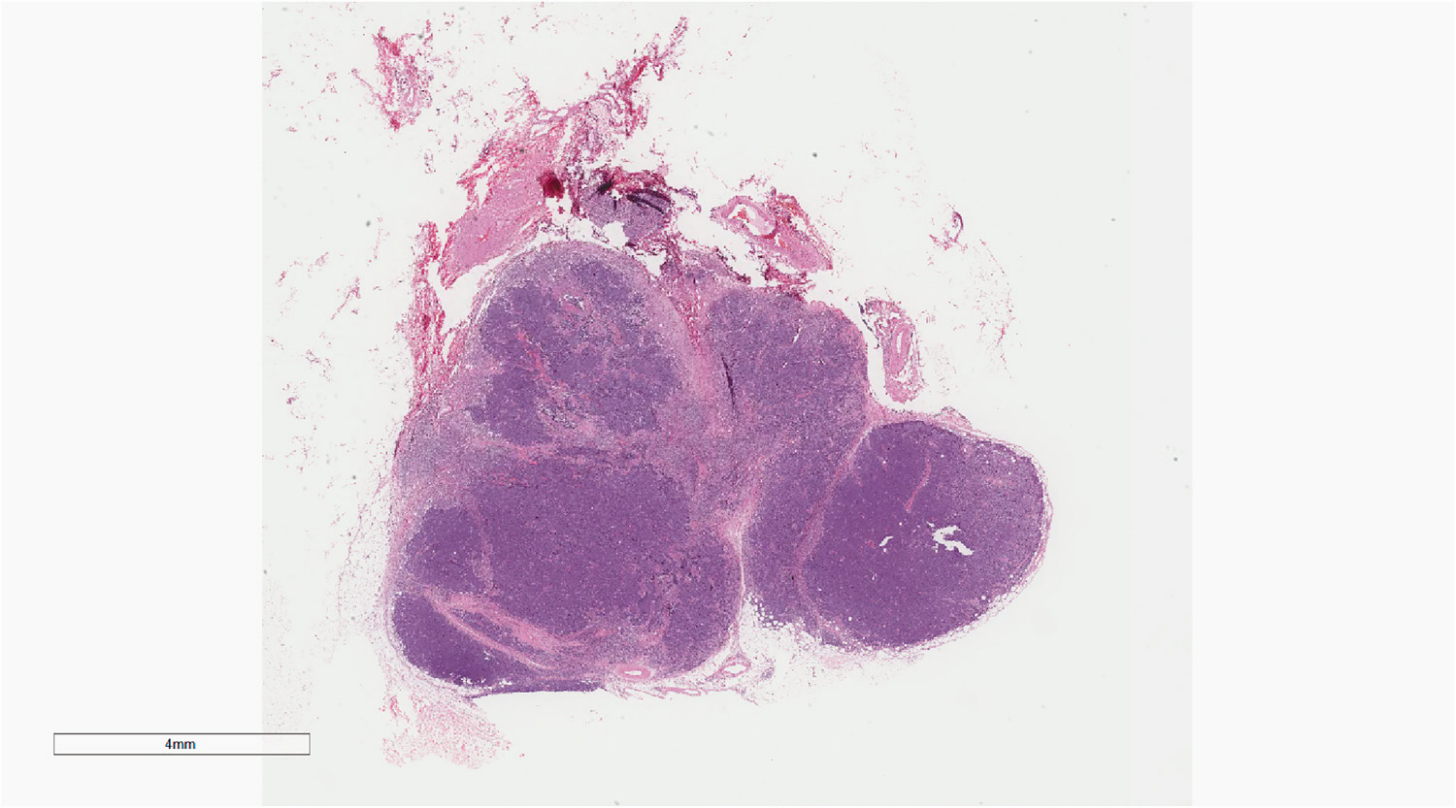
Under low magnification, tumor cells with nest-like masses were observed growing in the dermis. H&E ×4.

**Figure 3:**
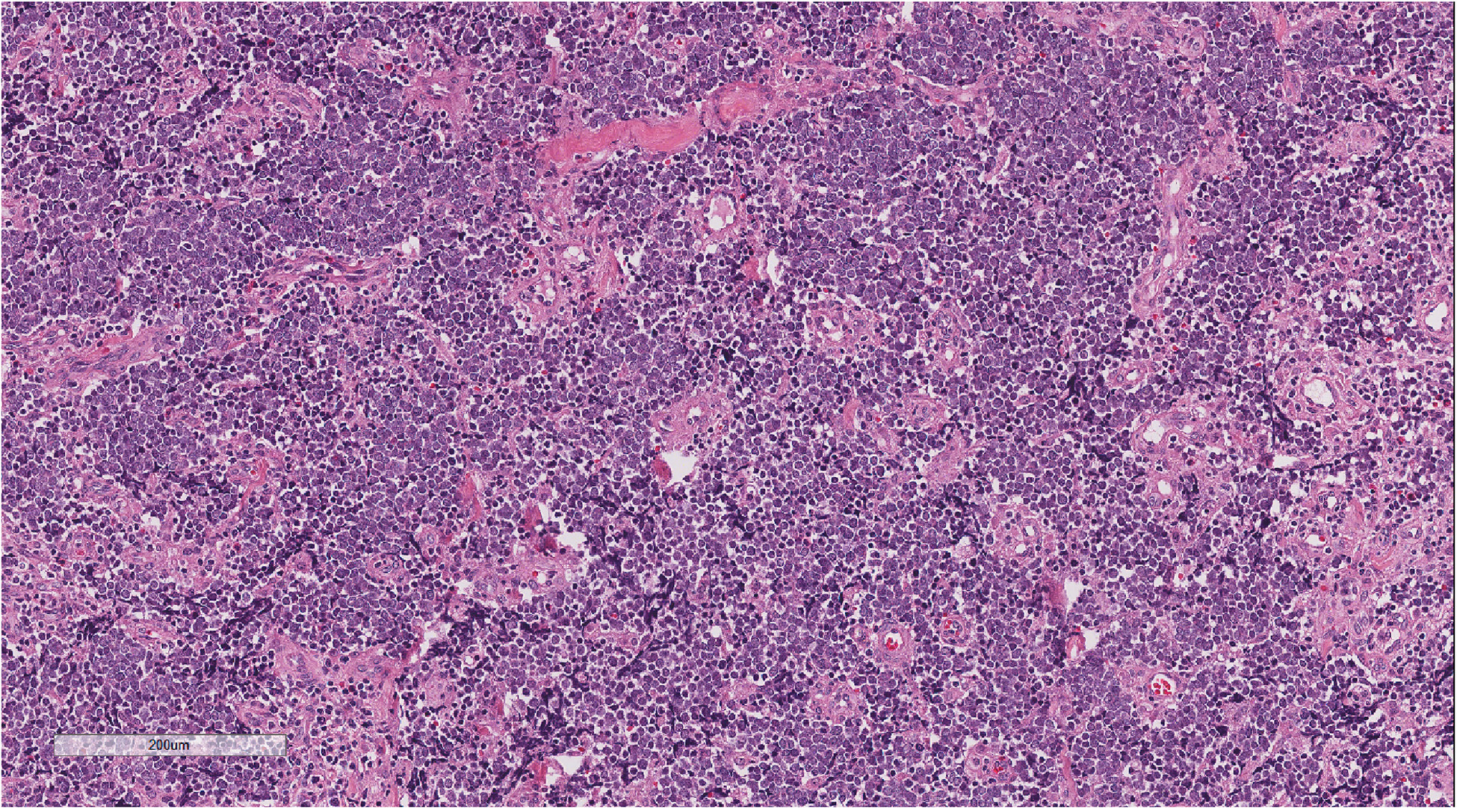
Moderate magnification demonstrated small, blue, and round tumor cells with diffuse invasive growth. H&E ×100.

**Figure 4:**
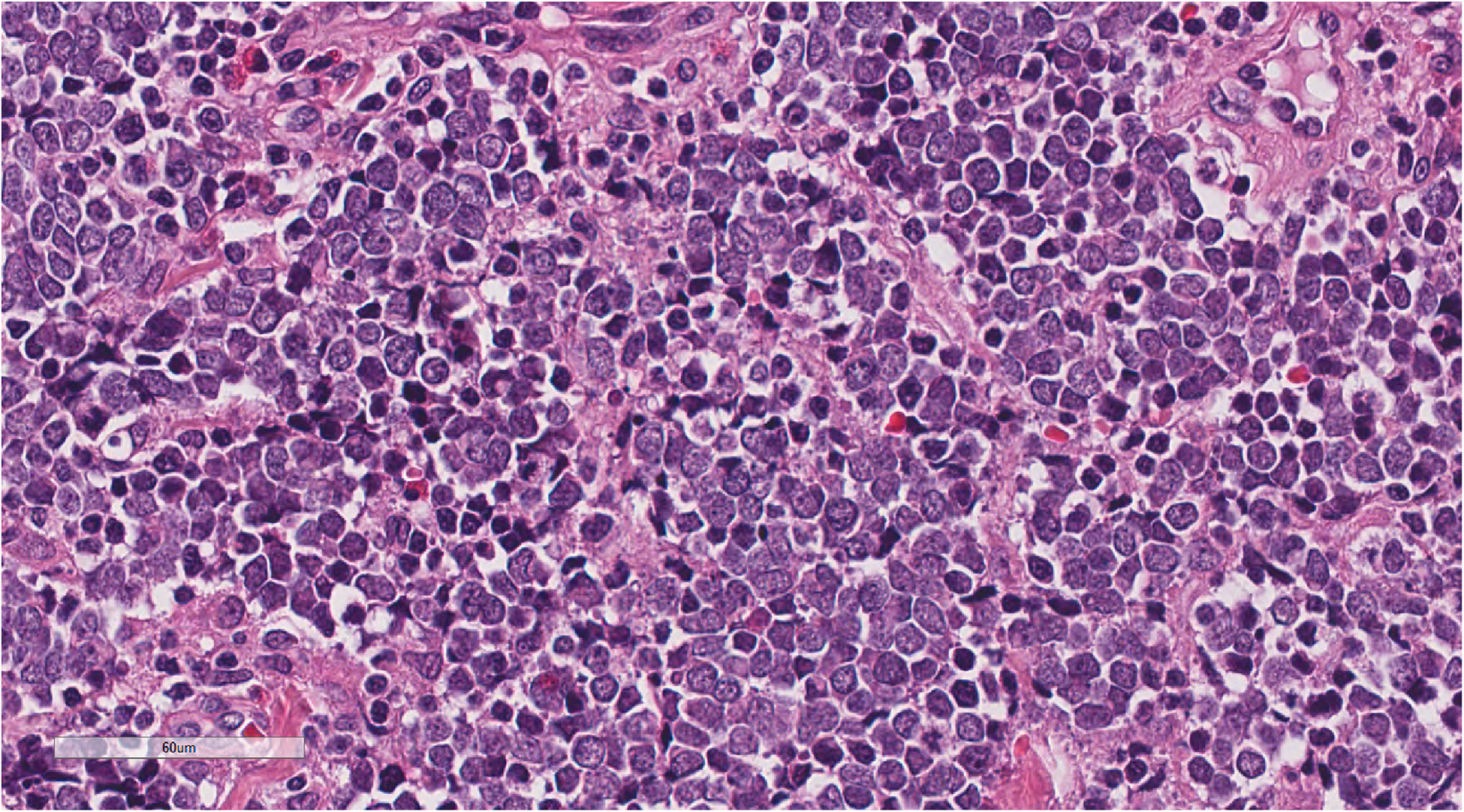
In high magnification, the tumor cells showed uniform size, sparse cytoplasm, round or oval nucleus, clear nuclear membrane, and fine chromatin. H&E ×400.

Microscopic examination of the three patients revealed similar morphological characteristics. Under low magnification, tumor cells with nest-like masses were observed growing in the dermis, and numerous red blood cells were detected in the stroma. Moreover, the surface squamous epithelium showed erosion and rupture, with focal hyperplasia and excessive keratosis of the peripheral squamous epithelium (Figure 2). Moderate magnification demonstrated small, blue, round tumor cells with diffuse invasive growth (Figure 3). In high magnification, the tumor cells showed uniform size, sparse cytoplasm, round or oval nucleus, clear nuclear membrane, fine chromatin, “salt and pepper,” and more nuclear division (Figure 4).

**Figure 5:**
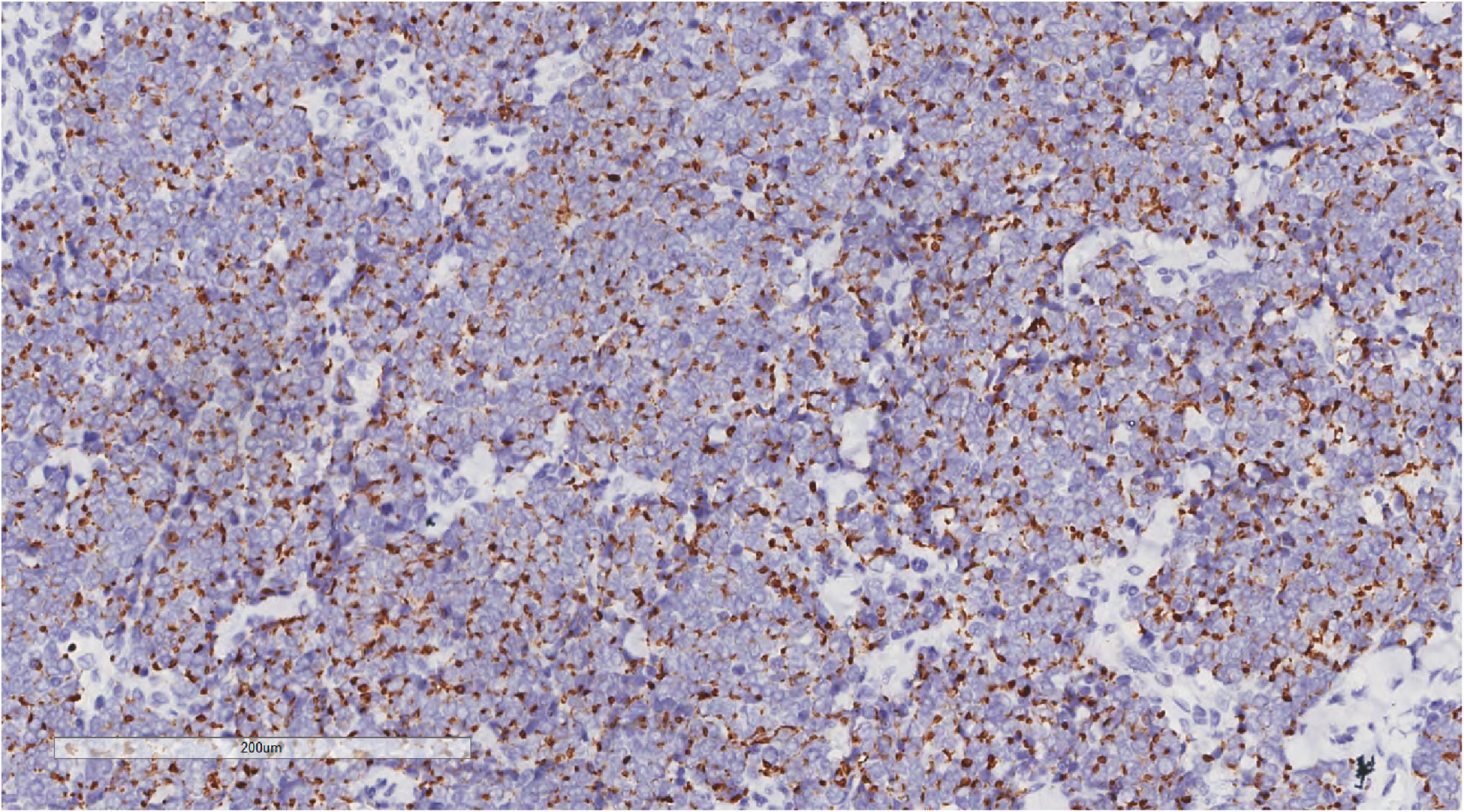
Immunohistochemistry reveals a tumor cell CK (nuclear punctate positivity, +). EnVision, ×200.

**Figure 6:**
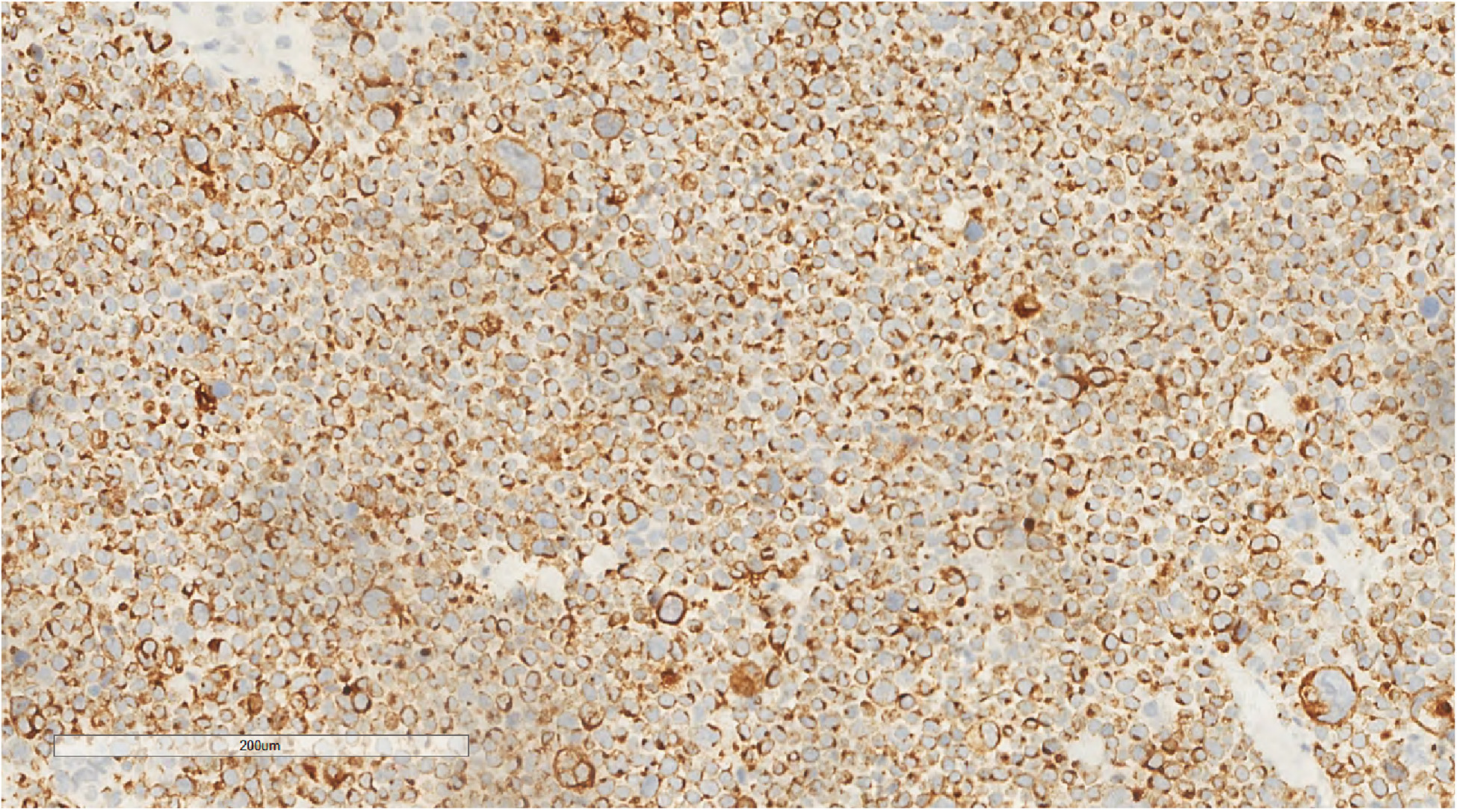
Immunohistochemistry reveals a tumor cell CK20 (nuclear punctate positivity, +). EnVision, ×200.

**Figure 7:**
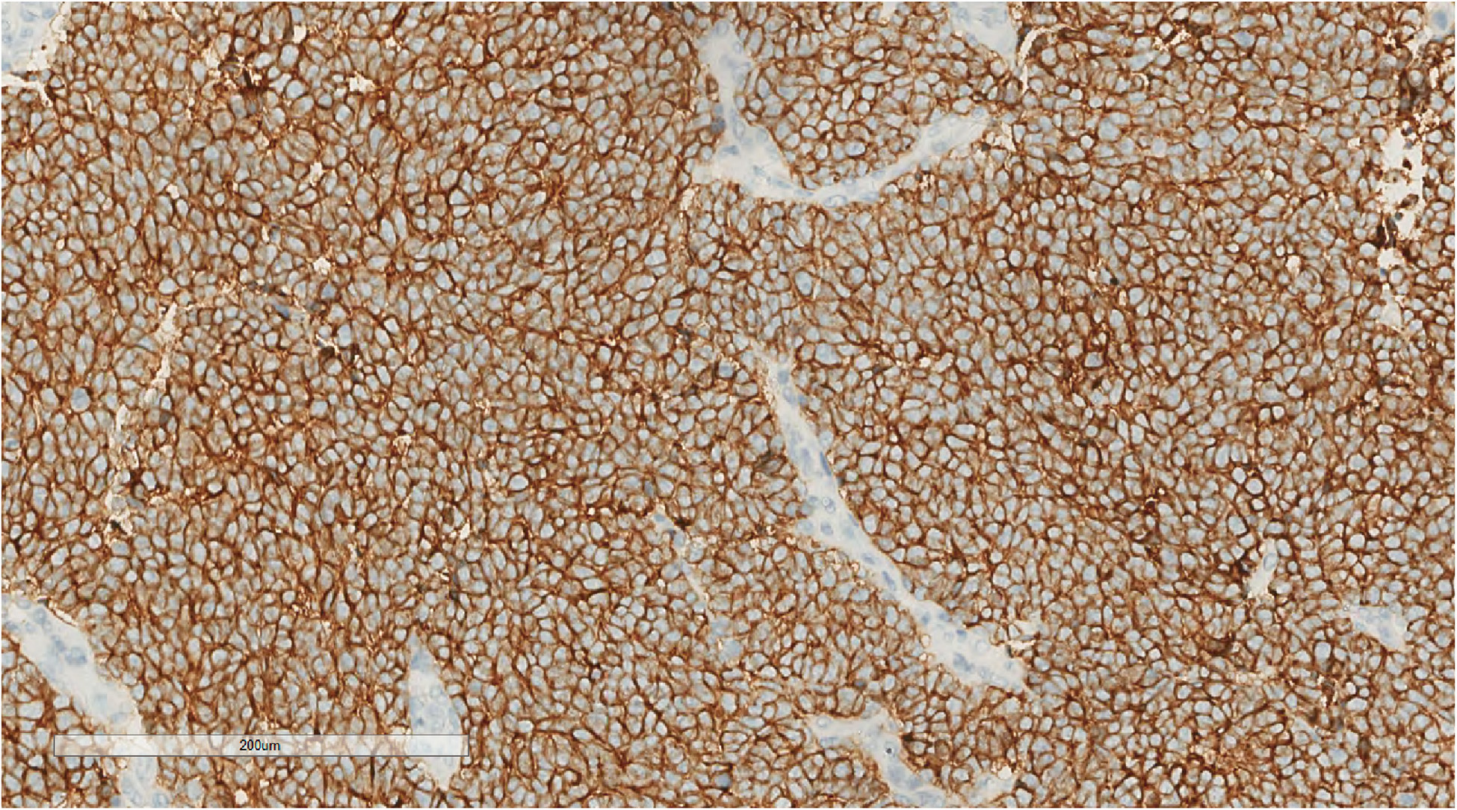
Immunohistochemistry reveals a tumor cell CD56 (+). EnVision, ×200.

Immunohistochemistry revealed one immunophenotype with nuclear punctate positivity for CK (Figure 5)/CK20 (Figure 6), two immunophenotypes with CK/CK20 negativity, similar residual immunophenotype, along with tumor cells that were EMA positive, CD56 positive (Figure 7), Syn positive, β-catenin membrane positive, p40, LCA, CD99, S100, HMB-45, SOX-10, TTF-1, CDX-2, and CD34 negative. The Ki-67 proliferation index of the tumor cells was 60–70%. Among the three cases of merkel cell carcinoma, 2 cases were negative for CgA immunolabeling and 1 case was positive.

## 4 Discussion

### 4.1 Clinical features of MCC

MCC occurs in middle-aged and older men, usually >70 years of age. Among our three patients, two were aged <70 years and one was >70 years. The clinical findings of MCCs are unspecific, with the tumors often located under the superficial surface of the body and frequently presenting as asymptomatic red or pink lesions and chronic sun exposure-induced skin damage that rapidly grows over weeks to months [8]. In one of our patients, a subcutaneous mass was detected 2 years after a wood stab wound in the left forearm, which subsequently increased quickly over a short period. Another patient presented with a skin rupture of the left toe 4+ months. The third patient had left eyelid lesions for 2 years. The rapid growth of MCCs is considered to be associated with the regulation [9]. MCC primarily occurs in the head, neck and limbs [8,10,11] trunk, and oral and genital mucosa in rare cases [8,12,13]. The MCCs of our three patients were located on the left forearm, left toe, and left eyelid. MCC is often misdiagnosed as inflammatory lesions, benign skin tumors, or other malignant tumors due to its non-specific and diverse clinical features [14]. Similarly, one of our patients was preliminarily diagnosed with lipoma, one with skin ulcer and infection, and one with squamous cell carcinoma.

A study by Heath et al. involving 195 patients outlined the most common clinical features of MCC to aid in its diagnosis. The authors suggested using the mnemonic AEIOU to summarize the salient features as follows: asymptomatic (asymptomatic/lack of tenderness), growth block (expanding rapidly), immunosuppression (immune suppression), older age than 50 years (older than 50 years), and exposure of light skin to UV rays (ultraviolet-exposed site on a person with light skin) [8]. In that study, 89% of the patients met at least three of the total five criteria, 32% fulfilled at least four criteria, and 6% satisfied all five criteria. Another study found that a low index of clinical suspicion and timely biopsy are crucial for diagnosing MCC [15].

### 4.2 Pathological features of MCC

A previous study reported that ocular MCC had an unclear boundary and infiltrative growth with no capsule. Further, the tumor diameter ranged from 0.2 to 20 cm, with most being less than 2 cm [8,16]. In the case of our three patients, the tumors were essentially consistent with a diameter range of 1.8–2.5 cm and a mean diameter of 2.1 cm. Microscopy examinations have shown that tumors are located in the dermis or subcutaneous tissue, without epidermis involvement in most cases. Moreover, these tumor cells appear to have a round or oval shape; relatively uniform size; dermis or subcutaneous tissue infiltration; and diffuse, trabecular, or nest-like distribution or several mixed patterns [17]. Electron microscopy studies have revealed the presence of cytoplasmic neurosecretory granules, cytoplasmic protrusions, and intermediate filaments around the nucleus of the tumor cells, thereby confirming Merkel cells as a progenitor line of MCC [18]. Immunophenotypic analysis has demonstrated that tumor cells express epithelial markers CK and CK20 and neuroendocrine markers CD56, Syn, and CgA. Although CK20 is a sensitive and specific marker of MCC, it typically produces small punctate paranuclear or cytoplasmic diffuse positive signals. However, a negative CK20 result does not exclude MCC diagnosis, as evidenced by up to 10–15% of MCC cases with CK20 negativity in previous literature [19]. With respect to our reported patients, two were negative for CK20, and one was positive for typical paranuclear CK20. Furthermore, biomarkers such as TTF-1 and CDX-2 can be used to identify metastatic MCC, whereas LCA, CD99, S100, HMB-45, and SOX-10 can exclude lymphoma, Ewing sarcoma, and malignant melanoma. A prior study has also indicated that microtubule-associated protein-2 is a sensitive and specific marker for diagnosing MCC [20]. The study also reported that tumor protein 63 (p63) expression was observed in 60% of MCC cases and was associated with a poor prognosis. However, additional data suggested that p63 cannot independently predict patient outcomes without considering the disease stage [21].

### 4.3 Molecular genetic characteristics of MCC

MCC is associated with multiple chromosomal abnormalities, including a short arm deletion of chromosome 1 (1p36), trisomy of chromosome 6, and a heterozygous loss of chromosome 13 [22]. The FHIT gene may be a potential candidate for tumor suppressor genes involved in MCC pathogenesis [23]. Additionally, the rearrangement of chromosome 5 is a common finding in MCCs. For example, an increase in chromosome 5p was detected in 32% of the patients with MCC, while a 5q12-21 deletion was found in 26% of the tumors [24]. Loss of heterozygosity on chromosome 10q23 has also been observed in MCC tumors [25]. Another investigation showed that a deletion of 13q14-21 in the RB1 tumor suppressor gene region was present in 26% of the tumors [26]. All these findings underline that the pattern of chromosomal abnormalities in MCC is scarcely consistent. Moreover, the multiple chromosomal abnormalities in MCC possibly reflect the highly aggressive biology of these tumors and imply that the related somatic aberrations may be sustained during the development of their malignant potential [22].

### 4.4 Imaging and laboratory examinations of MCC

MCC has been found to metastasize predominantly in the skin (28%) and lymph nodes (27%), followed by the liver (13%), lung (10%), bone (10%), and brain (6%) [16,27]. Therefore, imaging examination is particularly vital at this stage. Ultrasound can be employed to assess regional lymph node involvement. This imaging technique also provides live visualization with simultaneous fine or core needle biopsy and concise examination at a relatively lower cost [28]. High-resolution CT and MRI are still utilized to solve specific clinical problems. For instance, the CT imaging of the chest and abdomen can exclude metastatic lesions and primary sentinel lymph nodes, while MRI can image deep lymph nodes and subcutaneous fat nodular metastases [29]. In the case of MCC with bone metastasis, CT can show the metastasis as osteolysis or osteogenesis, and MRI can detect bone marrow involvement and extraosseous invasion. Although CT and MRI are effective methods, many centers have combined or replaced them with PET-CT [30]. One study revealed that PET-CT imaging resulted in changes in the stage classification among 33% of the patients and altered treatment strategies in 43% [31].

Previous studies have also found that most MCC occurrences are caused by the Merkel cell polyomavirus (MCPyV). Therefore, the quantitative detection of MCPyV oncoprotein antibodies can serve as a primary screen, while positive antibody titers in treated patients may indicate tumor recurrence. Analyzing blood samples of the tumor marker neuron-specific enolase can also assist in diagnosis [32]. It is a sign of the disease activity of neuroendocrine carcinomas (including MCCs) [25].

### 4.5 Differential diagnosis of MCC

Based on the morphology of the small, blue, round tumor cells, the differential diagnoses of MCC are as follows: (1) lymphoma: this cancer can be differentiated by immunohistochemistry due to its expression of LCA and PAX-5, whereas MCC does not express these two biomarkers. (2) Basal cell carcinoma (BCC): The periphery of BCCs exhibits tumor cell nests, which are not visible in MCCs. BCC also expresses Ber-EP4, while MCC has no such expression. (3) Malignant melanoma: Malignant melanomas, especially small-cell malignant melanomas, may be difficult to identify based on their morphological features. However, they can be distinguished from MCCs through the immunohistochemistry results (S-100, HMB-45, and Malan-A). In particular, S-100 may be expressed in MCC, but HMB-45 and Malan-A are not. (4) Ewing sarcoma: CD99 is a specific and sensitive marker of Ewing sarcoma, exhibiting strong expression on the membrane of Ewing sarcoma cells. Conversely, MCC cells do not express CD99. (5) Less differentiated squamous cell carcinoma: This subtype can be identified according to its immunohistochemistry staining findings. Less differentiated squamous cell carcinoma specifically expresses vimentin as well as CK and CK5/6 but not CK20, CK7, and neuroendocrine markers (including CD56, CgA, and Syn). In contrast, MCC expresses both the epithelial markers and the neuroendocrine markers. (6) Metastatic neuroendocrine carcinoma: These carcinomas are most distinguishable by their morphology and structure and can be detected by immunohistochemistry. Negative TTF-1 expression can exclude small-cell lung carcinoma and medullary thyroid carcinoma. Further, CDX-2 negativity can exclude neuroendocrine cancer of the intestine [33].

Finally, when the pathological diagnosis is MCC, tumor size, peripheral and resection margin status, lymphovascular invasion, and the involvement of bone/muscle/fascia or cartilage should be minimally included in the pathological report [11].

### 4.6 Treatment and prognosis of MCC

The prognosis of MCC is extremely poor, necessitating an aggressive treatment plan and possibly multimodal interventions. Many researchers suggest a combination of surgery, radiotherapy, and/or chemotherapy to mitigate the high risk of locoregional and/or metastatic recurrence [34–36]. Recently, immune checkpoint inhibitors have shown promise in treating metastatic disease. Specifically, avelumab, a monoclonal antibody targeting the programmed cell death-1 receptor ligand (PD-L1) approved by the National Institute of Health and Care Excellence, has shown encouraging survival outcomes [37].

MCC treatment is related to its clinical stage. A notable advantage in this staging system is the incorporation of lymph node assessment (clinical and pathological examination) as a factor in determining the stage, which is critical in MCC [15]. Surgical treatment can be helpful in early-stage MCC. In this context, international guidelines recommend that local lesions should be excised with a 12 cm margin, including the tissue down to myofascia, dabaost, or periosteum [38]. Additionally, surgery with local adjuvant radiotherapy was demonstrated to significantly ameliorate the condition of patients with stage I MCC, with the recommended adjuvant radiotherapy dose being 45–50 Gy [16]. Moreover, a study suggested that sentinel node biopsy should be performed in all patients diagnosed with MCC based on its findings that patients who were pathologically node-negative had a survival advantage over those who were clinically node-negative [39]. Early-stage MCC can potentially be cured by surgery and local radiotherapy. In contrast, patients with late MCC have a short median survival of only 6–9 months [40,41]. Chemotherapy is performed in MCC with metastatic progression primarily to relieve symptoms [42]. However, currently available data are insufficient to establish whether chemotherapy enhances the median survival in patients with advanced MCC [43]. Presently, no effective treatments exist for metastatic MCC outside of surgery and/or radiotherapy [2]. Immunotherapy with anti-programmed cell death 1 (PD-1) receptor/-PD-L1 antibodies has been found to be one of the most promising MCC treatments [43]. Among these, pembrolizumab is a humanized immunoglobulin G4 (IgG4) monoclonal anti-PD-1 antibody that has undergone phase II trials in patients with metastatic or recurrent regional MCC [44]. Avelumab is a fully human IgG1 anti-PD-L1 antibody that retains antibody-mediated cytotoxicity [45]. Avelumab was used as the first-line treatment in metastatic MCC, yielding an objective response rate of 62% [46]. Therefore, neo-adjuvant and/or adjuvant application of anti-PD-1/-PD-L1 therapy may be beneficial in patients with a high risk of postoperative MCC recurrence [47]. Another study suggested [48] that, given that approximately 80% of MCPyVs express viral proteins, integrating MCPyV vaccines into other immunotherapies may further improve their efficacy. This approach could be effective in treating advanced MCC and in preventing relapse following adjuvant immunotherapy.

There are some limitations to our study. First, this study is a single-center retrospective study, and second, the sample size is small and needs to be confirmed by a large sample study.

## 5 Conclusion

MCC is a highly malignant cutaneous neuroendocrine carcinoma, requiring the combination of pathological morphology examination and immunophenotyping to confirm the diagnosis. Moreover, the primary MCC treatment of surgical treatment should be supplemented with chemotherapy and/or local radiotherapy to alleviate its poor prognosis, easy recurrence or metastasis, and high mortality.
